# Significance of Neoadjuvant Downstaging in Carcinoma of Esophagus and Gastroesophageal Junction

**DOI:** 10.1245/s10434-020-08358-0

**Published:** 2020-03-21

**Authors:** S. K. Kamarajah, M. Navidi, S. Wahed, A. Immanuel, N. Hayes, S. M. Griffin, A. W. Phillips

**Affiliations:** 1 Northern Oesophagogastric Unit, Royal Victoria Infirmary, Newcastle University Trust Hospitals, Newcastle upon Tyne, UK; 2 Institute of Cellular Medicine, Newcastle University, Newcastle upon Tyne, UK; 3 School of Medical Education, Newcastle University, Newcastle upon Tyne, UK

## Abstract

**Objective:**

To determine the impact of downstaging on outcomes in esophageal cancer, the prognostic value of clinical and pathological stage, and the difference in survival in patients with similar pathological stages with and without neoadjuvant treatment.

**Background:**

There is little data evaluating adenocarcinoma and squamous cell carcinoma (SCC) and difference in outcomes for similar pathological stage with and without neoadjuvant treatment.

**Patients and Methods:**

Consecutive patients with esophageal cancer from a single center were evaluated. Patients with esophageal adenocarcinoma or SCC treated with transthoracic esophagectomy and two-field lymphadenectomy were included. Comparison of outcomes with those primarily treated with surgery was made. The cTNM and ypTNM 8th edition was used.

**Results:**

This study included 992 patients, of whom 417 received surgery alone and 575 received neoadjuvant therapy and surgery. In the neoadjuvant group, 7 (1%) had cTNM stage 2 and 418 (73%) had cTNM stage 3. Downstaging rates were similar between adenocarcinoma and SCC (54% vs. 61%, *p* = 0.5). Downstaging was associated with longer survival than patients with no change (adenocarcinoma, median: 82 vs. 26 months, *p* < 0.001; SCC, median: NR vs. 29 months, *p* < 0.001). On Cox regression analysis, downstaging was associated with significantly longer survival in adenocarcinoma but not in SCC. For SCC and more advanced adenocarcinoma, overall survival was significantly better when comparing like-for-like ypTN to pTN groups.

**Conclusions:**

Pathological stage provides a better estimate of prognosis compared with clinical stage. Downstaged patients may have an improved outcome over those with comparable pathological stage who did not receive neoadjuvant treatment.

**Electronic supplementary material:**

The online version of this article (10.1245/s10434-020-08358-0) contains supplementary material, which is available to authorized users.

Esophageal and gastroesophageal junction (GEJ) cancers are an aggressive disease affecting 450,000 people globally each year.^1^ Currently, neoadjuvant treatment followed by resection is the mainstay treatment of choice in locally advanced cancers, with multimodality approaches shown to improve outcomes.^2^^,^^3^ Neoadjuvant therapy improves long-term survival by providing locoregional disease control and reducing the risk of long-term recurrence.^4^ The most important prognostic factor of survival after neoadjuvant therapy followed by surgery is the burden of lymph node involvement. This is reflected in the most recent 8th edition of the American Joint Committee on Cancer (AJCC) and Union internationale contre le cancer (UICC) esophageal TNM staging.^5^^,^^6^ The current system grades N-stage from N0 to N3 depending on the number of nodes that are involved and has been updated to include clinical, pathological, and post-neoadjuvant staging classifications. Whilst its predecessors were based on patients who underwent treatment with surgery alone and did not receive neoadjuvant therapy,^6^^,^^7^ the current system uses data from those who have received neoadjuvant treatment,^5^^,^^6^ Neoadjuvant therapy has been shown to downstage the initial clinical staging (cTNM) of both tumor invasion (T) and nodal involvement (N).^8^–^10^

Historically, the initial clinical stage of patients with esophageal cancer was thought to determine the outcome.^11^ However, clinical staging is inaccurate, given that only a small proportion of patients will have similar pathological staging, especially in patients with cT3N3 disease.^12^–^14^ A recent study from this institution indicated nodal involvement in approximately 20% of patients who were originally staged as node negative. More recent evidence has indicated that disease stage following neoadjuvant therapy is a better prognostic marker in patients with adenocarcinoma^6^^,^^15^ than initial clinical stage. This has been reflected in the updated TNM staging system.^5^^,^^6^ The impact of neoadjuvant therapy on locoregional lymph nodes may therefore have an important bearing on prognosis and help identify patients who would benefit from further adjuvant treatment.

To further improve the current evidence base on the benefit of downstaging, this study aims to determine the impact of lymph node downstaging on the prognosis of patients with esophageal and junctional adenocarcinoma and squamous cell carcinoma (SCC) from a single high-volume center. Furthermore, this study aims to perform a stage-by-stage comparison of pathological staging in patients with (ypTNM) and without (pTNM) neoadjuvant therapy.

## Patients and Methods

### Patient Population

Consecutive patients treated for adenocarcinoma or squamous cell carcinoma of the esophagus or gastroesophageal junction between January 2000 and June 2017 from the Northern Oesophagogastric Unit, Newcastle upon Tyne were included. All patients were discussed at a multidisciplinary meeting and subsequently received neoadjuvant chemoradiotherapy followed by transthoracic esophagectomy. Patients were identified from a contemporaneously maintained database.

### Pretreatment Staging

All patients were staged according to standardized protocols which include endoscopy with biopsy, endoscopic ultrasonography, external ultrasonography of the neck (if required), and a thoracoabdominal computed tomography (CT) scan. A positron emission tomography (PET)/CT scan is used in patients being considered for radical (curative) treatment. In patients with histology proven locally advanced resectable malignancy without metastases [cT1N+ or cT3N0–3 or tumors of questionable resectability (cT4)], neoadjuvant chemoradiotherapy followed by surgery is the main treatment option. Patients with a histology other than adenocarcinoma or squamous cell carcinoma or with metastatic disease at the time of the operation were excluded.

### Treatment

Multiple neoadjuvant regimens were employed in the present study, determined by the standard of care and recruiting clinical trials at the time of treatment (Table 1). The majority of patients treated received neoadjuvant chemotherapy. Transthoracic esophagectomy with two-field lymph node dissection was performed within 5–8 weeks after completion of neoadjuvant therapy using a conventional or minimally invasive approach as previously described.^16^

**Table 1:** Demographics for adenocarcinoma and squamous cell carcinoma

	Adenocarcinoma				Squamous cell carcinoma			
	Downstaged	No change	Upstaged	*P* value	Downstaged	No change	Upstaged	*p* value
*n*	251	188	22		69	41	4	
Age at presentation (years)	64 [58, 69]	63 [56, 69]	60 [55, 68]	0.548	65 [58, 70]	65 [59, 68]	72 [69, 74]	0.094
Gender, male	220 (88)	162 (86)	18 (82)	0.705	31 (45)	20 (49)	2 (50)	0.917
ASA grade				0.422				0.388
Grade 1	34 (14)	30 (16)	2 (9)		14 (20)	2 (5)	0 (0)	
Grade 2	143 (57)	107 (57)	10 (45)		36 (52)	24 (59)	3 (75)	
Grade 3	64 (25)	40 (21)	9 (41)		17 (25)	13 (32)	1 (25)	
Grade 4	2 (1)	0 (0)	0 (0)					
Unknown	8 (3)	11 (6)	1 (5)		2 (3)	2 (5)	0 (0)	
Overall treatment				0.513				0.046
NAC + surgery	241 (96)	181 (96)	20 (91)		46 (67)	35 (85)	4 (100)	
NACRT + surgery	10 (4)	6 (3)	2 (9)		23 (33)	6 (15)	0 (0)	
NRT + surgery	0 (0)	1 (1)	0 (0)		–	–	–	
Surgical access, thoracic				0.081				0.351
Open	197 (78)	126 (67)	19 (86)		58 (84)	29 (71)	4 (100)	
Thoracoscopic	4 (1)	6 (3)	0 (0)		2 (3)	1 (2)	0 (0)	
Unknown	50 (20)	56 (30)	3 (14)		9 (13)	11 (27)	0 (0)	
Surgical access, abdomen				0.161				0.221
Open	203 (81)	134 (71)	19 (86)		61 (88)	32 (78)	4 (100)	
Laparoscopic	3 (1)	1 (1)	0 (0)		2 (3)	0 (0)	0 (0)	
Unknown	45 (18)	53 (28)	3 (14)		6 (9)	9 (22)	0 (0)	
Overall clinical staging				< 0.001				0.065
Stage 0	0 (0)	0 (0)	1 (5)		–	–	–	
Stage I	0 (0)	1 (1)	0 (0)		–	–	–	
Stage II	7 (3)	0 (0)	0 (0)		–	–	–	
Stage III	143 (57)	152 (81)	21 (95)		58 (84)	50 (98)	4 (100)	
Stage IV	101 (40)	35 (19)	0 (0)		11 (16)	1 (2)	0 (0)	
Tumor grade				0.005				0.06
Well	7 (3)	5 (3)	1 (5)		3 (4)	2 (5)	1 (25)	
Moderate	130 (52)	78 (41)	10 (45)		34 (49)	20 (49)	2 (50)	
Poor	97 (39)	103 (55)	11 (50)		14 (20)	16 (39)	1 (25)	
Unknown	17 (7)	2 (1)	0 (0)		18 (26)	3 (7)	0 (0)	
Lymph nodes examined	33 [25, 40]	35 [27, 44]	33 [30, 39]	0.08	29 [24, 37]	39 [29, 42]	32 [29, 34]	0.004
Margin status, R1	1 (0)	3 (2)	2 (9)	0.002	0 (0)	1 (2)	0 (0)	0.407
Lymphatic involvement, yes	98 (39)	136 (72)	16 (73)	< 0.001	11 (16)	22 (54)	4 (100)	< 0.001
Venous involvement, yes	65 (26)	98 (52)	16 (73)	< 0.001	6 (9)	21 (51)	4 (100)	< 0.001
Perineural involvement, yes	83 (33)	134 (71)	20 (91)	< 0.001	7 (10)	24 (59)	4 (100)	< 0.001
Tumor regression grade				< 0.001				0.001
1	12 (5)	0 (0)	0 (0)		12 (17)	0 (0)	0 (0)	
2	14 (6)	2 (1)	1 (5)		12 (17)	1 (2)	0 (0)	
3	53 (21)	18 (10)	0 (0)		9 (13)	4 (10)	0 (0)	
4	80 (32)	54 (29)	12 (55)		12 (17)	11 (27)	1 (25)	
5	17 (7)	10 (5)	4 (18)		2 (3)	7 (17)	2 (50)	
Unknown	75 (30)	104 (55)	5 (23)		22 (32)	18 (44)	1 (25)	
Extracapsular spread, yes	32 (13)	71 (38)	19 (86)	< 0.001	5 (7)	15 (37)	3 (75)	< 0.001

Note that pathological variables such as tumor grade, lymphatic involvement, venous involvement, and perineural involvement do not apply for those who had downstaged disease to pathological complete response (pCR)

### Pathology and Staging

Histopathological reporting was carried out by specialist gastrointestinal pathologists using a standardized proforma. This was in line with guidelines produced by the Royal College of Pathologists, which included tumor type and differentiation, depth of tumor infiltration, and tumor regression.^17^ Total number of nodes from each location and nodal metastases were recorded along with presence of extracapsular, lymphatic, venous, and perineural invasion.

Lymph nodes were dissected from specimen by the operating surgeon and analyzed separately by the pathologist.^18^ Pathological stage was determined using the AJCC 8th edition TNM staging system.^5^^,^^6^

### Comparison with Straight-to-Surgery Patients

In addition to investigating the impact of downstaging, a comparison on outcomes was carried out among patients who underwent surgery but did not receive neoadjuvant chemoradiotherapy. This included a cohort of patients who were pT2N0—neoadjuvant treatment is not routinely offered to patients with pT2N0 disease or earlier. Similar comparisons were made among patients who did not receive neoadjuvant treatment and had a more advanced pathological stage (pT3 N0 vs. ypT3 N0; pT3 N1 vs. ypT3 N1; pT3/4 N2/3 vs. ypT3/4 N2/3). These patients were discussed at the multidisciplinary meeting (MDM). Neoadjuvant treatment was declined due to concerns regarding fitness and potential for further deconditioning with neoadjuvant treatment. This could potentially exclude these patients from receiving curative-intent surgery. Further constraints such as inadequate renal and cardiac function excluded patients from receiving neoadjuvant oncological therapy based on local guidelines.

### Follow-Up and Definition of Recurrence

Patients were followed up until death or for 10 years. Patients were seen at 3–6-month intervals in the first 2 years, 6 monthly for 2 years, then annually. After 5 years, follow-up was on a yearly basis. Recurrence of disease was based on clinical grounds and confirmed endoscopically or radiologically.

### Definition of Downstaging

Patients were regarded as having been downstaged if the stage derived from analysis of the pathology specimen was earlier than the clinical stage. Stage movement was regarded as having occurred between any group (e.g., stage IVa to IIIb = 1 stage; stage IVa to IIIa = 2 stages).

### Statistical Analysis

Categorical variables were compared using the Chi squared test. Non-normally distributed data were analyzed using the Mann–Whitney *U* test. Survival was estimated using Kaplan–Meier survival curves and compared using the log-rank test. Multivariable analyses used Cox proportional hazards models. Stratified survival analyses by underlying histology (adenocarcinoma and squamous cell carcinoma) and by response to neoadjuvant therapy classification were performed. Analyses were also performed according to degree of downstaging (> 3 stages, 3 stages, 2 stages, and 1 stage). A *p* value of < 0.05 was considered to be statistically significant. Data analysis was performed using R Foundation Statistical software (R 3.2.2) with TableOne, ggplot2, Hmisc, Matchit, and survival packages (R Foundation for Statistical Computing, Vienna, Austria) as previously described.^17^

## Results

### Patient Characteristics

Between January 2000 and June 2017, 992 patients underwent resection for adenocarcinoma or squamous cell carcinoma of the esophagus. Of these, 575 patients received neoadjuvant therapy followed by a transthoracic esophagectomy [esophageal adenocarcinoma (*n* = 461); squamous cell carcinoma (*n* = 114)]. The remaining 417 patients received unimodality surgery, of whom 8 received adjuvant treatment. Rates of downstaging were higher in patients with SCC than adenocarcinoma, albeit not significantly so (61% vs. 54%, *p* = 0.5) (Table 1). Patients with SCC were more likely to receive neoadjuvant chemoradiotherapy (CRT) compared with adenocarcinoma (25% vs. 4%, *p* < 0.001) and less likely to have lymphatic invasion (32% vs. 54%, *p* < 0.001), venous invasion (27% vs. 39%, *p* = 0.028), and perineural invasion (31% vs. 51%, *p* < 0.001). Patients with SCC had significantly higher rates of TRG 1 compared with adenocarcinoma (11% vs. 3%, *p* < 0.001).

### Impact of Neoadjuvant Downstaging

Of patients with adenocarcinoma, 11% were downstaged by > 3 stages followed by 9% and 5% for 3 stages and 2 stages. Patients with downstaged tumors had higher rates of epirubicin, cisplatin, and capecitabine (ECX) chemotherapy regime than those with no change in tumor stage, although not significantly so (67% vs. 59%, *p* = 0.132). Downstaged patients were more likely to have lower rates of lymphatic (39% vs. 72%, *p* < 0.001), venous (26% vs. 52%, *p* < 0.001), and perineural involvement (33% vs. 71%, *p* < 0.001) and extracapsular spread (13% vs. 38%, *p* < 0.001) compared with those with no change.

Of patients with SCC, 18% were downstaged by > 3 stages followed by 4% and 20% for 3 stages and 2 stages. Patients receiving chemoradiotherapy had significantly higher rates of downstaged tumors compared with no change or upstaged (36% vs. 15% vs. 20%, *p* = 0.041). Downstaged patients were more likely to have lower rates of lymphatic (16% vs. 54%, *p* < 0.001), venous (9% vs. 51%, *p* < 0.001), and perineural involvement (10% vs. 59%, *p* < 0.001) and extracapsular spread (7% vs. 37%, *p* < 0.001) compared with those with no change.

### Overall Survival

**Fig. 1:**
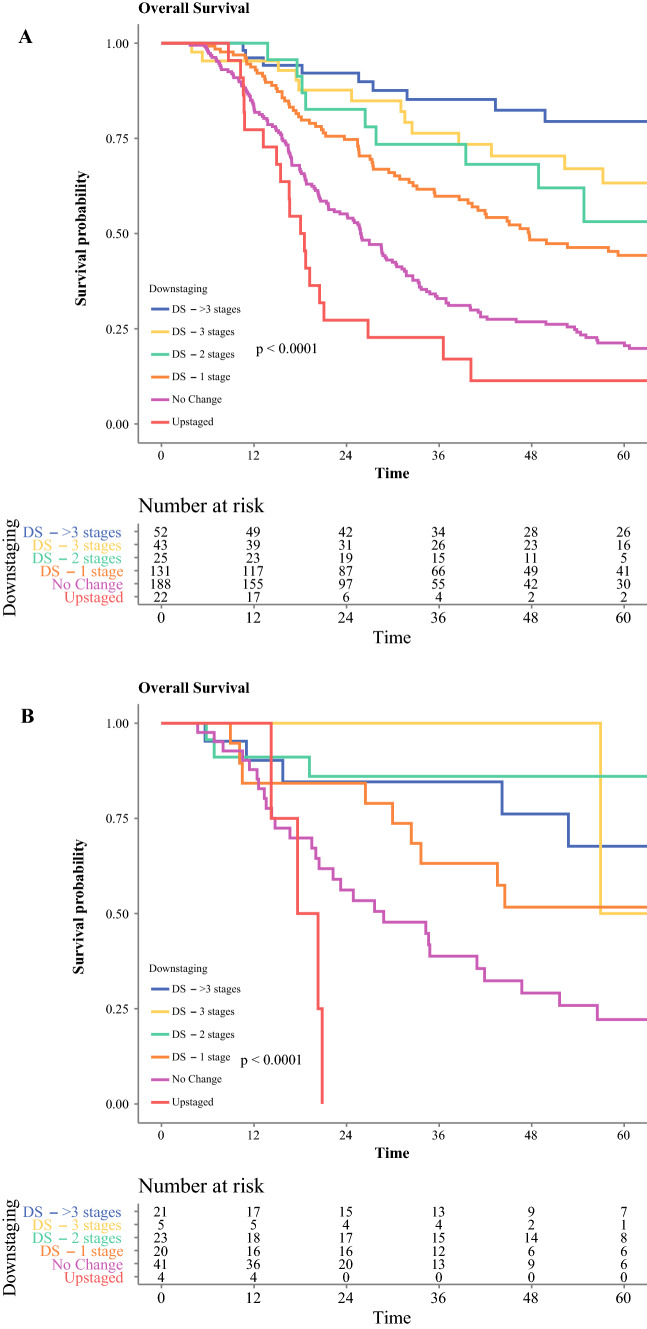
Overall survival of patients with esophageal cancer with neoadjuvant therapy: **a** adenocarcinoma and **b** squamous cell carcinoma

In patients with adenocarcinoma, downstaging was associated with significantly longer survival than no change (median: 82 vs. 26 months, *p* < 0.001). Patients who were downstaged by > 3 stages had a significantly longer survival than those downstaged by 3 stages vs. those by 2 stages vs. 1 stage (median: 170 vs. 148 vs. 77 vs. 48 months, *p* < 0.001) (Table 2; Fig. 1a).

**Table 2:** Overall survival of esophageal cancers for adenocarcinoma and squamous cell carcinoma

	Adenocarcinoma			Squamous cell carcinoma		
	*n*	Median survival, months	*p* value	*n*	Median survival, months	*p* value
All patients						
Downstaged > 3 stages	52	170.1 (100.7–NR)	< 0.001	21	90.6 (52.8–NR)	< 0.001
Downstaged 3 stages	43	147.7 (57.2–NR)		5	56.9 (56.9–NR)	
Downstaged 2 stages	25	76.9 (48.9–NR)		23	NR (NR–NR)	
Downstaged 1 stage	131	47.7 (39.8–85.4)		20	NR (33.6–NR)	
No change	188	25.9 (21.7–30.4)		41	28.8 (20.4–46.7)	
Upstaged	22	18.3 (15.4–26.8)		4	19.0 (14.2–NR)	
T2N0						
pT2N0	15	101.0 (61.6–NR)	0.5	4	70.7 (49.6–NR)	0.001
ypT2N0	42	148.0 (74.1–NR)		21	NR (NR–NR)	
T3N0						
pT3N0	27	71.0 (40.4–163.0)	0.2	19	42.1 (13.8–NR)	0.3
ypT3N0	86	105.0 (59.2–NR)		23	67.5 (52.8–NR)	
T3/4 N1						
pT3/4 N1	87	23.7 (16.7–34.7)	0.5	37	17.1 (13.5–29.2)	0.048
ypT3/4 N1	109	25.8 (20.4–32.6)		33	34.3 (23.2–115.7)	
T3/4 N2/3						
pT3/4 N2/3	21	15.9 (12.5–24.8)	0.018	3	11.5 (7.6–NR)	0.2
ypT3/4 N2/3	84	25.8 (20.6–31.2)		15	20.3 (14.23–46. 7)

In patients with SCC, downstaging was associated with significantly longer survival than no change (median: NR vs. 29, *p* < 0.001). Patients downstaged by > 3 stages had a significantly longer survival than those downstaged by 3 stages vs. 2 stages vs. 1 stage (median: 91 vs. 57 vs. NR vs. NR months, *p* < 0.001) (Table 2; Fig. 1b).

For adenocarcinoma, the multivariable adjusted Cox regression analysis revealed that nonresponder (HR: 1.79, 95% CI 1.34–2.40, *p* < 0.001), lymphatic involvement (HR: 1.45, 95% CI 1.06–1.98, *p* = 0.021), perineural involvement (HR 1.57, 95% CI 1.18–2.09, *p* = 0.002), and extracapsular spread (HR 1.75, 95% CI 1.28–2.39, *p* < 0.001) were adverse prognostic factors for overall survival. For squamous cell carcinoma, there were no independent prognostic factors for survival. The Cox regression models are presented in Supplementary Table 1.

### Subgroup Analysis by Neoadjuvant Therapy

To determine the impact of downstaging of neoadjuvant therapy, a stage-by-stage comparison between patients who received neoadjuvant treatment and those neoadjuvant naïve was carried out. During this period, 417 patients did not receive neoadjuvant therapy, of whom 5% were pT2N0 (*n* = 19), 11% were pT3N0 (*n* = 46), 29% were pT3/4 N1 (*n* = 124), and 6% were pT3/4 N2/N3 (*n* = 24).

**Fig. 2:**
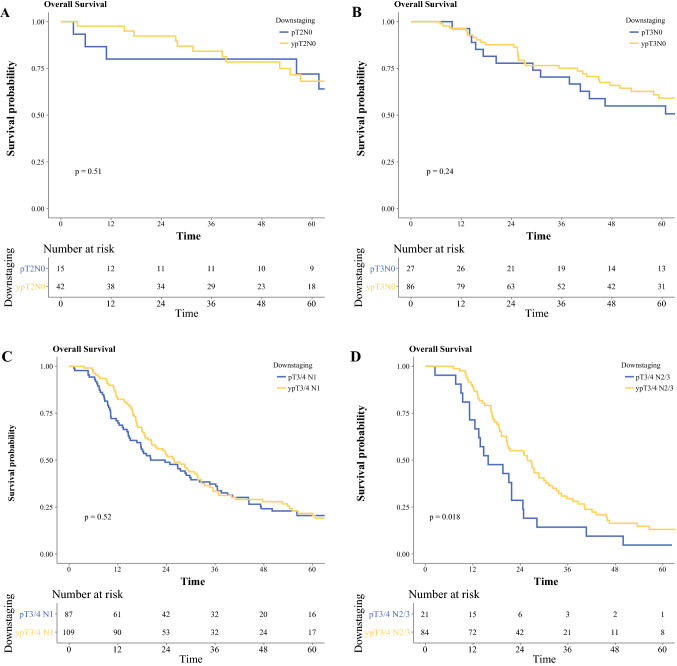
Stage-by-stage overall survival of patients with esophageal adenocarcinoma with and without neoadjuvant therapy: **a** T2N0, **b** T3N0, **c** T3/4 N1, and **d** T3/4 N2/3

In patients with adenocarcinoma, there were no significant differences in survival in patients with and without neoadjuvant therapy for T2N0, T3N0, and T3/4 N1 (Fig. 2a–c). However, median survival was significantly longer for ypT3/4 N2/3 compared with pT3/4 N2/3 (median 26 vs. 16 months, *p* = 0.018, Fig. 2d).

In patients with SCC, median survival was significantly longer for ypT2N0 compared with pT2N0 (median: NR vs. 71 months, *p* = 0.001) and for ypT3/4 N1 compared with pT3/4 N1 (median: 34 vs. 17 months, *p* = 0.048).

### Subgroup Analysis by Downstaging of T3/4 N+

**Fig. 3:**
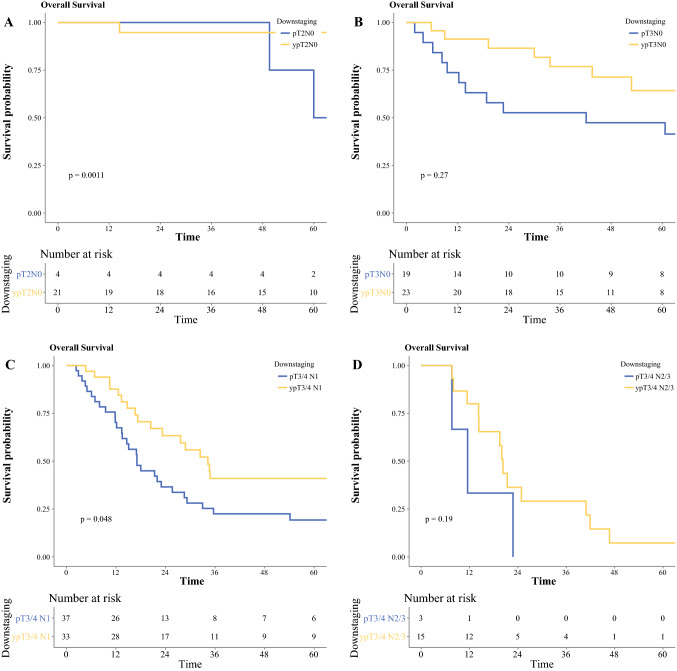
Stage-by-stage overall survival of patients with esophageal squamous cell carcinoma with and without neoadjuvant therapy: **a** T2N0, **b** T3N0, **c** T3/4 N1, and **d** T3/4 N2/3

Supplementary Figs. 1–4 present a more detailed survival analysis of patients initially staged at cT3/4 N + who received chemotherapy stratified for adenocarcinoma and squamous cell carcinoma and who were downstaged to ypT0N0 (Supplementary Fig. 1), ypT1/2 N0 (Supplementary Fig. 2), ypT1/2 N + (Supplementary Fig. 3), or ypT3/4 N0 (Supplementary Fig. 4). In each survival graph, the two control curves represent stage-matched patients who were not administered neoadjuvant chemotherapy (pTNM) and patients who were not downstaged by chemotherapy (i.e., nonresponders who were still ypT3/4 N + after surgical resection). In all of these survival analyses, a significant survival benefit was seen in chemotherapy responders versus nonresponders while no difference was observed between responders and stage-matched controls (Fig. 3).

## Discussion

Esophageal cancer is an aggressive disease with a high recurrence rate of over 50% post-neoadjuvant therapy and surgery.^19^^,^^20^ These results corroborate what has been found in other recent studies,^15^^,^^21^ that disease downstaging with neoadjuvant treatment is associated with better overall survival. This has led to the acceptance of neoadjuvant therapy for locally advanced esophageal cancer as the standard of care.^22^–^24^ However, it is apparent that not all patients respond to neoadjuvant treatment to the same degree, and the observed impact could be used to tailor adjuvant treatment. In addition, there appeared to be better survival in patients who received neoadjuvant therapy compared with similarly staged neoadjuvant-naïve patients. This was apparent through all the stages for squamous cell carcinoma and evident in more advanced adenocarcinoma.

Current staging of esophageal cancer uses the AJCC 8th edition. This now includes clinical, pathological, and post-neoadjuvant stage groupings, allowing their application to clinical practice to be examined. These results highlight the importance of stage post-neoadjuvant treatment for predicting outcomes. To allow accurate staging postsurgery, it is important that adequate lymphadenectomy be carried out.^5^^,^^6^ The new staging recognizes a disparity between the lymphadenectomy required to help accurately stage the disease and the lymphadenectomy required for oncological clearance. In the former, this is dependent on tumor size, with short cancers (< 2.5 cm) requiring up to 60 nodes and cancers greater or equal to 2.5 cm requiring 20 nodes.^25^ To contrast this, the number of nodes required for oncological clearance may be related to the depth of tumor invasion, with T1 tumors needing at least 10 nodes, T2 tumors 20 nodes, and T3 > 30 nodes.^26^ A two-field lymphadenectomy was routinely performed, which ensured that the stage of disease is likely to be accurate with a high median yield in this study (33, range 10–98).^27^

In this study, patients with adenocarcinoma saw a stepwise decline in survival associated with downstaging by more than 3 stages through to those who were upstaged. Patients with a 3-stage improvement had a 5-year survival of 60%, while those showing a 1–2-stage improvement had a prognosis of over 36%. In contrast, patients in whom the disease apparently remained static or deteriorated with neoadjuvant treatment had 5-year survival of only 21% and 13%, respectively. This impact was less pronounced in the squamous cell cohort. This was particularly noticeable after 36 months had passed for SCC patients. Whilst SCC patients who were upstaged or had no change from their clinical stage fared very poorly, initial benefits that seemed apparent disappeared as time from treatment progressed in those with apparent downstaging. It is difficult to determine why this may have happened, but one possibility is a poorer systemic effect for those receiving chemoradiotherapy. This may provide excellent local disease control but have a reduced impact on treating micrometastases.

Approximately 55% of those with adenocarcinoma and 62% with squamous cell carcinoma demonstrated significant improvement between clinical stage and posttreatment stage. Overall, over 80% of patients had adenocarcinoma. Neoadjuvant treatment was generally chemotherapy (95%) in these patients. Chemoradiation treatment was used in 28% of patients with SCC, but only 5% of those with adenocarcinoma were associated with complete pathological response, approaching 30% in some studies, with a further 20% demonstrating significant partial tumor response.^28^ This would reflect the findings from this study, where a significantly higher degree of TRG1 was seen in SCCs, which might reflect the higher proportion receiving chemoradiotherapy. The CROSS study, which evaluated chemoradiation prior to esophagectomy, demonstrated a 23% complete pathological response,^2^ such levels for chemotherapy prior to surgery have not been reached.^29^–^32^ Whilst complete response was not evaluated in this study, no significant difference in stage improvement was noted between the use of chemoradiation and chemotherapy. Equally, histological stage had no influence on the degree of downstaging that occurred. Complete pathological response for patients with adenocarcinoma was 4% compared with 14% for SCC. Meanwhile, the SANO^33^ and Esotrate-Prodige^34^ studies seek to determine the impact of watchful waiting in these patients. Previous data have suggested that outcomes of those downstaged are poorer than those with the equivalent “early” disease,^35^ however, these data suggest that this may not be the case. Comparison of outcomes between pTNM and ypTNM demonstrated a significant improvement in survival for patients who received neoadjuvant treatment who were T2 N0 for SCC but not for adenocarcinoma. This impact was also evident for both adenocarcinoma and SCC in the most advanced cancers seen (T3/4 N2/3).

There are a number of limitations to this study that need to be addressed. Comparison of pathological stage with post-neoadjuvant stage may falsely represent the impact of neoadjuvant treatment. Whilst the presumption is that stage migration is due to the effect of neoadjuvant treatment, it is difficult to gauge the full impact given the inherent inaccuracies with staging modalities.^36^ Further, as this study was not a randomized trial, it is potentially susceptible to bias. This is particularly true when comparing outcomes between pathological stage and those who received neoadjuvant chemotherapy. However, overall outcomes represent an unselected consecutive number of patients from a high-volume unit. These patients, though, passed the same MDM and received a standardized two-stage two-field transthoracic esophagectomy, providing a level of quality assurance which some retrospective collaborative studies lack. It appears that both chemotherapy and chemoradiotherapy have a similar impact in downstaging disease and that downstaging confers a definite survival benefit, although this may be different between SCC and adenocarcinoma.

This study reinforces the understanding that post-neoadjuvant stage influences prognosis. Whilst some may advocate “complete” restaging prior to progressing to surgery, induction therapy is known to potentially impact on the reliability of staging modalities.^32^ It may also be important to consider the post-neoadjuvant stage when deciding on the merit of adjuvant treatment. There has been some suggestion that adjuvant treatment may confer some benefit in node-positive patients with adenocarcinoma who received neoadjuvant chemoradiotherapy,^37^ and it already forms part of the standard of treatment in patients receiving the MAGIC protocol chemotherapy.^11^

Neoadjuvant oncological therapy with surgery improves survival over surgical intervention alone. Multiple randomized trials have reinforced the superiority of multimodal therapy over surgery alone.^11^^,^^38^^,^^39^ However, recent studies have demonstrated a deleterious impact of neoadjuvant oncological therapy on fitness.^40^ The impact of this decline on survival has not been established. However, the ability to predetermine the impact of neoadjuvant therapy on disease stage based on specific biological factors of a tumor may further individualize oncological therapy. This may spare a subgroup of patients the adverse physiological impact of neoadjuvant oncological therapy,^41^ in whom, as identified by this paper, neoadjuvant therapy leads to static or worsening stage with no apparent improvement in survival.

In conclusion, this study demonstrates the significant impact of downstaging in patients with both SCC and adenocarcinoma. The optimal neoadjuvant treatment for both remains controversial. Understanding and assessment of response to chemotherapy are imperative if patients are to receive individualized treatment.

## Electronic supplementary material

Below is the link to the electronic supplementary material.

Supplementary material 1 (DOCX 470 kb)
