# Supplementary material for: Significance of Neoadjuvant Downstaging in Carcinoma of Esophagus and Gastroesophageal Junction
Source: Ann Surg Oncol. 2020 Mar 21;27(9):3182–92. doi: 10.1245/s10434-020-08358-0 (PMC7410857; doi:10.1245/s10434-020-08358-0)
Supplement: Supplementary file 1 — Supplementary material 1 (DOCX 470 kb) [file 10434_2020_8358_MOESM1_ESM.docx]

Supplementary Table 1 Multivariable Cox Regression model of overall survival for oesophageal adenocarcinoma and squamous cell carcinoma

|  | Adenocarcinoma |  | SCC |  |
| --- | --- | --- | --- | --- |
|  | HR (CI95%) | p-value | HR (CI95%) | p-value |
| Age at Presentation | 1.01 [1.00, 1.03] | 0.117 | 1.00 [0.96, 1.03] | 0.857 |
| Gender, Male | 1.30 [0.89, 1.90] | 0.168 | 1.43 [0.73, 2.81] | 0.296 |
| ASA Grade |  |  |  |  |
| Grade 1 |  |  |  |  |
| Grade 2 | 0.98 [0.68, 1.41] | 0.914 | 0.87 [0.33, 2.27] | 0.776 |
| Grade 3 | 1.02 [0.68, 1.52] | 0.934 | 0.56 [0.19, 1.66] | 0.295 |
| Grade 4 | 1.59 [0.33, 7.59] | 0.558 | 0.32 [0.05, 2.03] | 0.228 |
| Unknown | 0.90 [0.47, 1.71] | 0.745 | 2.63 [0.57, 12.20] | 0.217 |
| Tumour Grade |  |  |  |  |
| Well |  |  |  |  |
| Moderate | 1.36 [0.62, 2.96] | 0.441 | 4.22 [0.82, 21.72] | 0.085 |
| Poor | 1.68 [0.77, 3.68] | 0.191 | 3.26 [0.56, 18.90] | 0.188 |
| Unknown | 0.72 [0.19, 2.69] | 0.628 |  |  |
| Longitudinal Margin, R1 | 1.30 [0.55, 3.07] | 0.544 | - | - |
| Lymphatic Involvement, Yes | 1.45 [1.06, 1.98] | 0.021 | 0.90 [0.39, 2.11] | 0.817 |
| Venous Involvement, Yes | 1.02 [0.76, 1.38] | 0.875 | 1.68 [0.66, 4.27] | 0.278 |
| Perineural Involvement, Yes | 1.57 [1.18, 2.09] | 0.002 | 1.36 [0.56, 3.28] | 0.493 |
| Tumour Regression Grade |  |  |  |  |
| Grade 1 |  |  |  |  |
| Grade 2 | 0.45 [0.10, 2.00] | 0.292 | 2.62 [0.36, 18.80] | 0.339 |
| Grade 3 | 0.52 [0.15, 1.85] | 0.312 | 0.83 [0.08, 8.21] | 0.87 |
| Grade 4 | 0.76 [0.22, 2.62] | 0.667 | 1.79 [0.28, 11.53] | 0.539 |
| Grade 5 | 0.87 [0.24, 3.16] | 0.833 | 2.74 [0.37, 20.02] | 0.321 |
| Unknown | 0.71 [0.21, 2.44] | 0.591 | 1.61 [0.28, 9.36] | 0.599 |
| Extracapsular Spread, Yes | 1.75 [1.28, 2.39] | <0.001 | 1.38 [0.63, 3.04] | 0.422 |
| Node Disssection, Two-Field | 0.35 [0.08, 1.48] | 0.155 | 0.10 [0.01, 1.07] | 0.057 |
| Tumour Response |  |  |  |  |
| Downstaging |  |  |  |  |
| No Change | 1.79 [1.34, 2.40] | <0.001 | 2.29 [0.97, 5.42] | 0.059 |
| Up-staged | 2.17 [1.26, 3.73] | 0.005 | 4.18 [0.88, 19.83] | 0.072 |

Supplementary Figure 1 Comparison of surgically treated patients with esophageal cancers, downstaged from cT3/4 N+ to ypT0N0. Control groups are represented by cT3/4 N+ to ypT3/4N+ (not downstaged) and T0N0 (no chemotherapy) (A) Adenocarcinoma (B) Squamous cell carcinoma

B

A

|  | n | Median Survival, months | p-value | n | Median Survival, months | p-value |
| --- | --- | --- | --- | --- | --- | --- |
| pT0N0 | 9 | 124.9 (76.9 NR) | <0.001 | 5 | NR NR NR) | 0.004 |
| ypT0N0 | 11 | 170.1 (NR NR) |  | 13 | 90.6 90.6 NR) |  |
| ypT3/4 N+ | 187 | 24.8 (20.5 - 28.6) |  | 52 | 23.2 20.3 40.9) |  |

Supplementary Figure 2 Comparison of surgically treated patients with esophageal cancers, downstaged from cT3/4 N+ to ypT1/2 N0. Control groups are represented by cT3/4 N+ to ypT3/4 N0 (not downstaged) and T1/2 N0 (no chemotherapy). (A) Adenocarcinoma (B) Squamous cell carcinoma

B

A

|  | n | Median Survival, months | p-value | n | Median Survival, months | p-value |
| --- | --- | --- | --- | --- | --- | --- |
| pT1/2 N0 | 160 | 138.6 103.7 NR | <0.001 | 22 | 81.4 49.6 NR | <0.001 |
| ypT1/2 N0 | 51 | 147.7 74.1 NR |  | 26 | NR NR NR |  |
| ypT3/4 N+ | 187 | 24.8 20.5 28.6 |  | 52 | 23.2 20.3 40.9 |  |

Supplementary Figure 3 Comparison of surgically treated patients with esophageal cancers, downstaged from cT3/4 N+ to ypT1/2 N+. Control groups are represented by cT3/4 N+ to ypT3/4 N+ (not downstaged) and T1/2N+ (no chemotherapy). (A) Adenocarcinoma (B) Squamous cell carcinoma

B

A
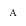

|  | n | Median Survival, months | p-value | n | Median Survival, months | p-value |
| --- | --- | --- | --- | --- | --- | --- |
| **pT1/2 N+** | 33 | 46.5 (31.5 - NR) | 0.002 | 19 | 60.6 (16.0 - NR) | 0.013 |
| **ypT1/2 N+** | 24 | 47.7 (26.4 - NR) |  | 4 | NR (NR - NR) |  |
| **ypT3/4 N+** | 187 | 24.8 (20.5 - 28.6) |  | 52 | 23.2 (20.3 - 40.9) |  |

Supplementary Figure 4 Comparison of surgically treated patients with esophageal cancers, downstaged from cT3/4 N+ to ypT3/4 N0. Control groups are represented by cT3/4 N+ to ypT3/4 N0 (not downstaged) and T3/4 N0(no chemotherapy) (A) Adenocarcinoma (B) Squamous cell carcinoma

B

A
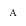


|  | n | Median Survival, months | p-value | n | Median Survival, months | p-value |
| --- | --- | --- | --- | --- | --- | --- |
| **pT3/4 N0** | 28 | 71.0 (40.4 - 163.4) | <0.001 | 22 | 41.3 (18.8 - NR) | 0.038 |
| **ypT3/4 N+** | 187 | 24.8 (20.5 - 28.6) |  | 52 | 23.2 (20.3 - 40.9) |  |
| **ypT3/4 N0** | 70 | 170.5 (59.2 - NR) |  | 22 | 64.1 (33.6 - NR) |  |
